# Virtual Reality for Screening of Cognitive Function in Older Persons: Comparative Study

**DOI:** 10.2196/14821

**Published:** 2019-08-01

**Authors:** Sean Ing Loon Chua, Ngiap Chuan Tan, Wei Teen Wong, John Carson Allen Jr, Joanne Hui Min Quah, Rahul Malhotra, Truls Østbye

**Affiliations:** 1 Duke-NUS Medical School Singapore Singapore; 2 SingHealth Polyclinics Singapore Singapore; 3 Duke University Durham, NC United States

**Keywords:** virtual reality, feasibility studies, mental status and dementia tests, technology, video games, dementia, cognitive dysfunction

## Abstract

**Background:**

The prevalence of dementia, which presents as cognitive decline in one or more cognitive domains affecting function, is increasing worldwide. Traditional cognitive screening tools for dementia have their limitations, with emphasis on memory and, to a lesser extent, on the cognitive domain of executive function. The use of virtual reality (VR) in screening for cognitive function in older persons is promising, but evidence for its use is sparse.

**Objective:**

The primary aim was to examine the feasibility and acceptability of using VR to screen for cognitive impairment in older persons in a primary care setting. The secondary aim was to assess the module’s ability to discriminate between cognitively intact and cognitively impaired participants.

**Methods:**

A comparative study was conducted at a public primary care clinic in Singapore, where persons aged 65-85 years were recruited based on a cut-off score of 26 on the Montreal Cognitive Assessment (MoCA) scale. They participated in a VR module for assessment of their learning and memory, perceptual-motor function, and executive function. Each participant was evaluated by the total performance score (range: 0-700) upon completion of the study. A questionnaire was also administered to assess their perception of and attitude toward VR.

**Results:**

A total of 37 participants in Group 1 (cognitively intact; MoCA score≥26) and 23 participants in Group 2 (cognitively impaired; MoCA score<26) were assessed. The mean time to completion of the study was 19.1 (SD 3.6) minutes in Group 1 and 20.4 (3.4) minutes in Group 2. Mean feedback scores ranged from 3.80 to 4.48 (max=5) in favor of VR. The total performance score in Group 1 (552.0, SD 57.2) was higher than that in Group 2 (476.1, SD 61.9; *P*<.001) and exhibited a moderate positive correlation with scores from other cognitive screening tools: Abbreviated Mental Test (0.312), Mini-Mental State Examination (0.373), and MoCA (0.427). A receiver operating characteristic curve analysis for the relationship between the total performance score and the presence of cognitive impairment showed an area under curve of 0.821 (95% CI 0.714-0.928).

**Conclusions:**

We demonstrated the feasibility of using a VR-based screening tool for cognitive function in older persons in primary care, who were largely in favor of this tool.

## Introduction

### Background and Rationale

Dementia is becoming more prevalent worldwide. About half of the dementia cases are in Asia, and the total number of cases worldwide is forecasted to increase to 63 million in 2030 [1]. In Singapore, one in ten people aged ≥60 years may have dementia [2]. Patients with dementia demonstrate significant cognitive decline in at least one or more cognitive domains, including complex attention, executive function, learning and memory, language, perceptual-motor function, and social cognition [3]. Mild cognitive impairment (MCI) represents a “middle ground” between normal ageing and dementia, and there has been growing interest in its timely diagnosis [4]. Although the Mini-Mental State Examination (MMSE) is the most widely applied test for dementia screening, the Montreal Cognitive Assessment (MoCA) is considered the best alternative for screening of MCI [5].

Besides the MMSE and MoCA, there are more than 40 other tests available for cognitive screening in health care settings [5]. A challenge with the application of commonly used paper-and-pencil or even digitalized screening tools is the limited cognitive domains that they assess. This is at the expense of other cognitive domains like executive function [6] and perceptual-motor function, which, when deficient, are associated with a high risk of progression to dementia [7]. Furthermore, the scoring in many of these screening tools is influenced by factors such as education level and cultural background [8]. Functional status scales used to assess the severity of dementia, such as the Barthel Index for Activities of Daily Living (ADL), also heavily depend on subjective observational measures. One plausible solution to overcome some of these limitations is the employment of virtual reality (VR) technology.

Virtual reality is a technology that provides interaction between a user and artificially generated environments. In recent years, with technological advancement, the use of VR has become more widespread. Beyond entertainment purposes, VR has also found purpose within certain fields of medicine [9] such as cognitive rehabilitation and training [10]. As a screening tool, VR has shown to have greater ecological validity [11], which reflects how well these tests predict real-world settings. This is in contrast with the contrived testing environment around the routine screening tests used today.

Several studies have attempted to investigate the use of VR to screen for cognitive impairment in older persons. Tong et al [12] used a tablet technology to screen for abnormal cognitive status in the emergency department, while Zygouris et al [13] developed a cognitive training app and compared its performance with established neuropsychological tests. Neither of these studies applied VR to cognitive screening in primary care, which is the most relevant setting for early detection of cognitive impairment [14].

### Study Aims

The primary aim of this study was to examine the feasibility and acceptability of using VR to screen for cognitive impairment in older persons in a primary care setting. The VR platform used in this study is a new tool (referred to as the RE@CH assessment module or VR module) developed by the Institute of Technical Education (ITE), College West, Singapore. Feasibility is defined by the proportion of participants who successfully complete the RE@CH assessment module within a stipulated time. Acceptability is based on the feedback received at the end of the study with regard to acceptance, perception, and experience with VR.

The secondary aim was to assess the ability of the RE@ACH assessment module to discriminate between cognitively intact and cognitively impaired participants. Performance was assessed by first establishing a scoring algorithm on the module, followed by comparing the performance scores between the two groups. Validity was assessed by examining the correlation of the scores against other routine cognitive screening assessments including the Abbreviated Mental Test (AMT) [15], MMSE [16], and MoCA [17]. This was followed by receiver operating characteristic (ROC) curve analysis to determine a useful cut-off score.

## Methods

### Study Design

This study was conducted at SingHealth Polyclinics-Outram, a public primary care clinic located in the central region of Singapore and serving a multiethnic Asian population.

Most patients attend these polyclinics to visit their primary care physicians or family physicians for management of their noncommunicable diseases such as hypertension, dyslipidemia, and type 2 diabetes mellitus. Subspecialized clinics operate on selected days of the month at this study site, such as the GeRiAtric serviCE (GRACE) memory clinic, which screens patients for suspected cognitive impairment including MCI and dementia.

### Participants and Eligibility

**Figure 1:**
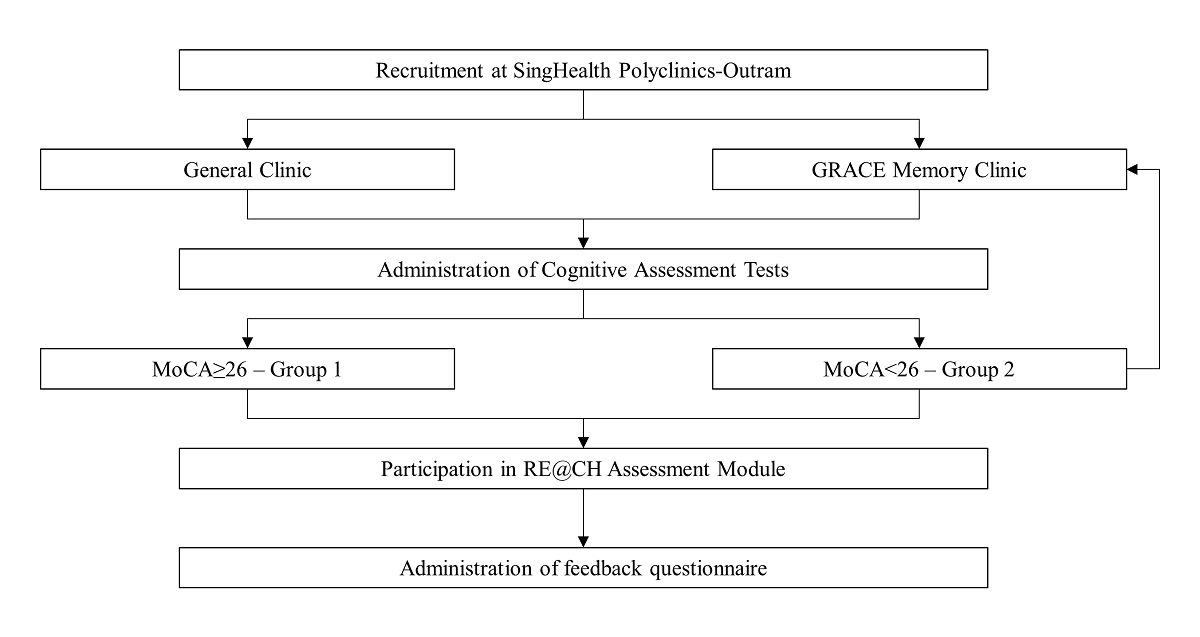
Study flow. GRACE: GeRiAtric serviCE; MoCA: Montreal Cognitive Assessment.

The participants were older persons registered at SingHealth Polyclinics-Outram, aged between 65 and 85 years. Sixty participants were targeted based on a small to medium standardized effect size [18] of the secondary aim (difference between mean performance scores and correlation coefficient), calculated using the upper confidence limit approach [19]. Of the 137 participants who were approached, 60 (43.8%) were recruited and enrolled after providing consent. All were recruited directly at the study site from March 2019 to April 2019 and were assessed by either the primary investigator (PI) or co-PI of the study project. Potential participants were patients waiting to see the doctor in the general clinic or GRACE memory clinic (Figure 1). They were either screened selectively at the waiting area or referred by physicians from those clinics.

All participants were required to understand the procedure for using the RE@CH assessment module, perform the movements involved in the study, and have the mental capacity to provide written informed consent. Those with poor vision, inability to follow verbal commands, aphasia, impairment of kinetic abilities that could inaccurately affect their performance on the assessment module, and unwillingness or inability to comply with the study protocol were excluded. Those with severe functional impairment based on the Lawton instrumental ADL (iADL) scale [20] were also excluded.

Enrolled participants provided written informed consent before entering the study, and this included consent to access their electronic medical records. All participants had an acceptable mental capacity, and consent was obtained in the presence of a witness. The MoCA cognitive assessment screen was performed and participants were divided into two groups (Figure 1), each meeting specific eligibility criteria:

- Group 1: Cognitively intact individuals, as defined by a MoCA score of ≥26. Those with pre-existing formal diagnosis of cognitive impairment of any degree or a history of cerebrovascular accident or neurological deficits were excluded.
- Group 2: Cognitively impaired individuals, as defined by a MoCA score of <26.

### RE@CH Assessment Module

**Figure 2:**
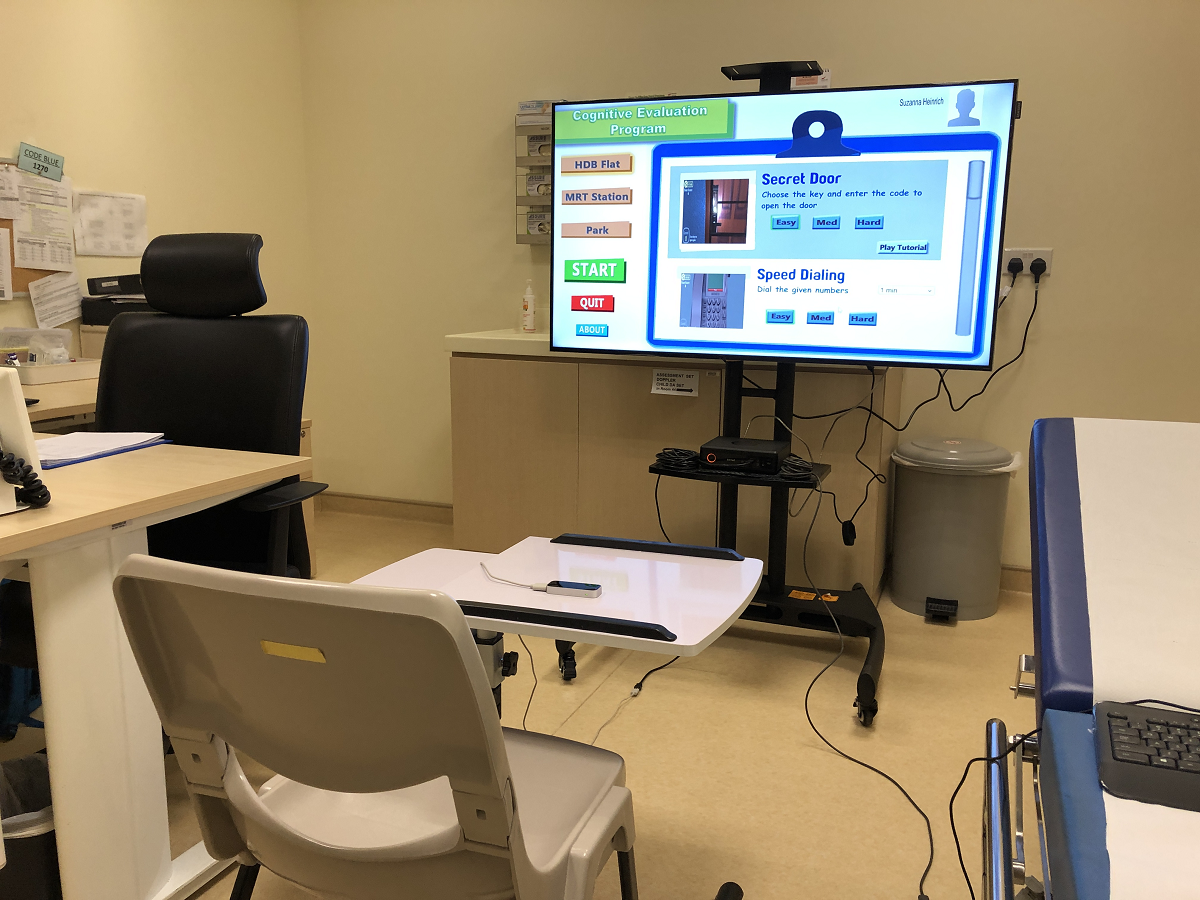
Setup of the RE@CH assessment module in the consultation room.

**Figure 3:**
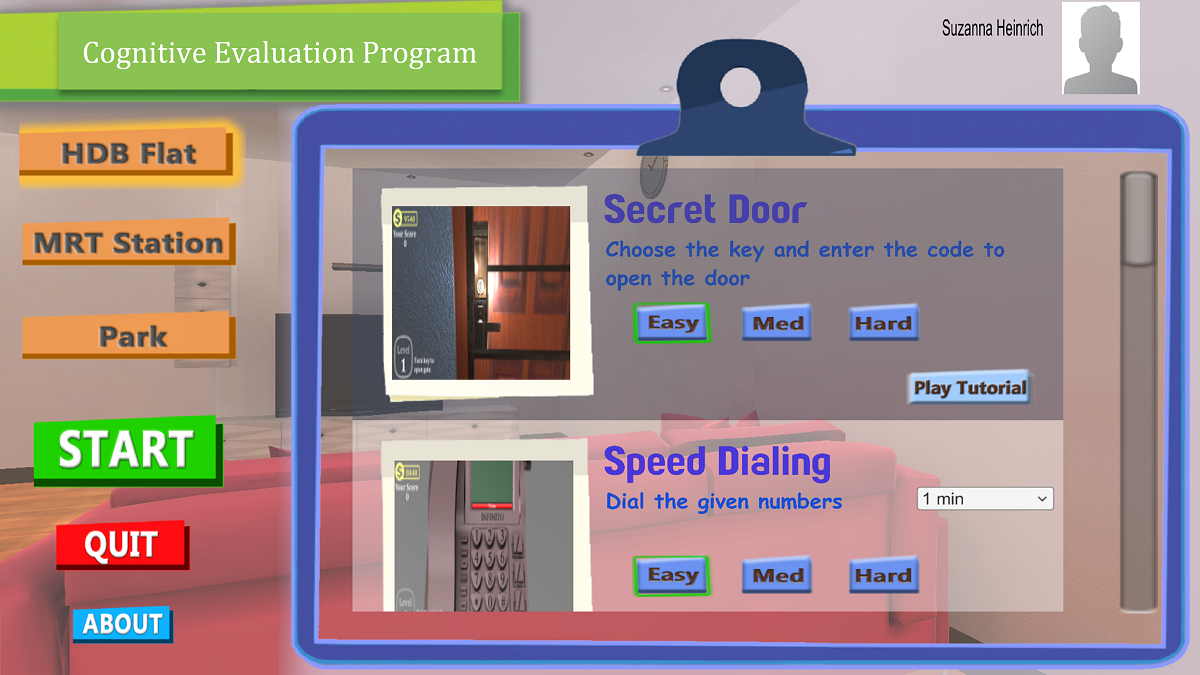
Main screen page of the RE@CH assessment module, showing two 3D games: Secret Door (opening a door using the right key) and Speed Dialling (making a phone call by recalling a predefined 8-digit number).

The RE@CH assessment module (Figure 2) uses VR and motion sensor (Leap motion) technology to replicate activities encountered in daily living as 3D games (Figure 3). The virtual environment was projected on a 55-inch 2D screen that recreates an immersive experience for the user. Through these activities, several cognitive domains were assessed. These included learning and memory, perceptual-motor function, and executive function, which had the greatest emphasis.

The study team designed a scoring algorithm (Table 1) to appraise the participant’s performance on the RE@CH assessment module. Seven relevant tasks were selected and the scored depending on the participants’ ability to complete the task correctly within the stipulated time, the number of attempts, and the proportion of tasks performed correctly.

Participants had to complete various tasks via the module using appropriate hand gestures. Before the formal VR assessment, they were guided through one orientation task to familiarize themselves with the mechanics of the system. Their performance on the next seven key activities was scored manually using the scoring algorithm (Table 1) in the following order:

1. Opening a door using the correct key and passcode number
2. Making a phone call by recalling a predefined 8-digit number
3. Identifying: (a) Famous people, (b) Advertisement of groceries, and (c) 4-digit number on a lottery slip on a newspaper
4. Sorting household objects according to category
5. Picking an outfit appropriate for a specified occasion
6. Withdrawing cash from an automated teller machine
7. Shopping for groceries in a provision shop

Prior to each task, the study team guided the participant on the requirement of the task through a short tutorial.

**Table 1:** Scoring algorithm on RE@CH assessment module.

Cognitive domain, task, content		Score					Remarks
		0	25	50	75	100	
**Perceptual-Motor**								
	1—Opening door with correct key and passcode number	No attempt	Unable to complete Step 1	Complete Step 1	Complete Step 1 and 2	Complete Steps 1, 2, and 3	Step 1: Pick the correct key Step 2: Open the door Step 3: Key in the correct number
**Learning and Memory**								
	2—Make a phone call, recalling the 8-digit number in predefined sequence	No attempt	Unable to complete on 3 attempts	Complete on 3 attempts	Complete on 2 attempts	Complete on 1 attempt	Every reattempt after a wrong digit is keyed in, is counted as 1 attempt
**Executive Function**								
	3—Identify items in each category from the newspaper: (1) famous people, (2) advertisement of groceries, and (3) 4-digit number on a lottery slip	No attempt	Fail to identify any category correctly	Identify 1 category correctly	Identify 2 categories correctly	Identify all 3 categories correctly	1 item in from each category
	4—Housekeeping: Sort things inside the room	No attempt	Sort 0 items correctly	Sort 1 item correctly	Sort 2 items correctly	Sort all 3 items correctly	Sorting 1 round of 3 items according to their appropriate category
	5—Dressing/grooming: Pick appropriate outfit for occasion	No attempt	Pick appropriate outfit for 0 of 3 tries	Pick appropriate outfit for 1 of 3 tries	Pick appropriate outfit for 2 of 3 tries	Pick appropriate outfit for all 3 tries	Picking the appropriate outfit for 3 different occasions
	6—Handling finances: Withdrawing cash from ATM^a^	No attempt	Unable to complete Step 1	Complete Step 1	Complete Step 1 and 2	Complete Steps 1, 2, and 3	Step 1: Insert ATM card Step 2: Enter correct PIN^b^ Step 3: Select and withdraw correct amount
	7—Handling finances: Shopping at provision shop	No attempt	Unable to complete Objective 1 with 0 correct item	Complete Objective 1 with 1 correct item	Complete Objective 1 with 2 or more correct items	Complete Objective 1 and 2	Objective 1: Pick 3 correct items Objective 2: Pay the correct amount

^a^ATM: automated teller machine.

^b^PIN: personal identification number.

### Data Collection

First, AMT, MMSE, and MoCA cognitive screening tests were carried out in order and scored as part of the cognitive function assessment to determine eligibility and classification into either of the two groups.

Next, baseline characteristics were collected, including demographics, functional status (Barthel Index and Lawton iADL), clinical parameters, and past medical history. All data were obtained directly from the participants, except for their past medical history, which was from their electronic medical records.

Subsequently, the VR component of the study began once the PI or co-PI of the study started the briefing of the participant on the module and ended after the completion of the seventh activity on the RE@CH assessment module. The start and end time were recorded and used to calculate the participant’s time spent on the module, which included the time spent on the tutorial and explanation by the facilitator.

Finally, the participants completed an interviewer-administered questionnaire to assess their perception toward VR (Textbox 1). There were six questions, and the answers were rated on a Likert scale (1 - strongly disagree, 2 - disagree, 3 - neutral, 4 - agree, 5 - strongly agree). Total feedback scores were calculated by summing the scores from the six questions.

**Sample questionnaire assessing subjects’ perception toward virtual reality experience, categorized by individual questions.**

1. The VR software I have experienced is easy to use.
2. The amount of time I spent with the VR software is acceptable to me.
3. The use of VR software helps to make the experience in the clinic more interactive.
4. The use of VR technology to help diagnose medical condition appeals to me.
5. I would not mind seeing more new technologies being used by the doctor/medical staff during the consultation.
6. Overall, I enjoyed the VR experience in the clinic.

### Outcome Measures

To achieve the primary aim of examining the feasibility and acceptability of the VR module, the following outcomes were measured: percentage of participants who successfully completed the RE@CH assessment module, time taken to complete the RE@CH assessment module, and scores from the feedback questionnaire.

To achieve the secondary aim of assessing the performance and validity of the RE@CH assessment module, the following outcomes were measured: performance scores on the RE@CH assessment module and scores on other cognitive assessment tests including AMT, MMSE, and MoCA. Additional statistical analysis was performed to derive the correlation between these outcome measures and to obtain the ROC curve.

All outcome measures were recorded in both study groups.

### Statistical Analysis

Baseline characteristics were compared between the groups using a two-sample *t* test and the Fisher exact test for continuous and categorical variables, respectively. Summary statistics were calculated individually for the recruitment statistics and questionnaire-dependent feedback scores. Groups 1 and 2 were compared using the two-sample *t* test.

Performance scores were analyzed at each task level, given a total score, and compared between the two groups using the methods described above, as appropriate. Correlation between performance scores and other variables was assessed using Pearson correlation. A logistic regression was performed based on the total performance scores, followed by ROC analysis to assess its predictive capability to discriminate between cognitively intact and cognitively impaired individuals.

A *P* value<.05 was considered statistically significant (two-sided). Analyses were performed using SAS University Edition software (Version 9.4M6 of the SAS System for Windows; SAS Institute Inc, Cary, NC).

## Results

### Sample Description

Based on their MoCA scores, 37 participants with a score≥26 were placed in Group 1 (cognitively intact) and 23 participants with a score<26 were placed in Group 2 (cognitively impaired). Of those in Group 2, 10 were new cases pending referral to the GRACE memory clinic. Baseline characteristics of participants in both groups were compared (Table 2). There were no statistically significant differences in age and gender between the two groups. Mean cognitive assessment scores, based on AMT, MMSE and MoCA, were all higher in Group 1 than in Group 2.

**Table 2:** Baseline characteristics by cognitive status.

Characteristics		Cognitively intact (n=37)	Cognitively impaired (n=23)	*P* value^a^
Age (years), mean (SD)		70.7 (3.6)	73.2 (5.4)	.06
**Gender, n (%)**				.21
	Female	26 (70.3)	15 (65.2)	
	Male	11 (29.7)	8 (34.8)	
**Years of education, n (%)**				<.001
	<6	4 (10.8)	12 (52.2)	
	6-10	23 (62.2)	8 (34.8)	
	>10	10 (27.0)	3 (13.0)	
**Functional status scores, mean (SD)**				
	ADL^b^	98.5 (2.6)	98.3 (3.9)	.78
	iADL^c^	22.8 (0.5)	21.7 (1.4)	.002
**Cognitive assessment scores, mean (SD)**				
	AMT^d^	9.6 (0.6)	8.3 (1.3)	<.001
	MMSE^e^	28.8 (1.0)	25.9 (3.4)	<.001
	MoCA^f^	27.8 (1.2)	22.2 (3.3)	<.001

^a^In categories where the mean values can be directly compared, the *P* values are given individually. In categories where the distribution across subcategories are compared, only *P* values for the main categories are given.

^b^ADL: activities of daily living, maximum score of 100.

^c^iADL: instrumental activities of daily living, maximum score of 23.

^d^AMT: Abbreviated Mental Test, maximum score of 10.

^e^MMSE: Mini-Mental State Examination, maximum score of 30.

^f^MoCA: Montreal Cognitive Assessment, maximum score of 30.

### Primary Aim: Feasibility and Acceptability

#### Recruitment Statistics and Time to Completion

All 60 (100%) enrolled participants successfully completed the study. The mean total time spent on the RE@CH assessment module was not significantly different between the two groups (Group 1: 19.1 [SD 3.6] minutes; Group 2: 20.4 [SD 3.4] minutes; *P*=.17; Table 3).

#### Assessment of Perception Toward Virtual Reality

Results from the questionnaire were favorable toward the VR experience. Mean feedback scores for each question (over a scale of 1-5) ranged from 3.80 to 4.48 (Table 3). The largest proportion of responses to all statements was “Agree” or “Strongly agree.” The difference in total feedback scores between the study groups was not statistically significant (*P*=.62; Table 3).

**Table 3:** Total time spent on the RE@CH assessment module and feedback scores between study groups.

Factor			Cognitively intact (n=37)		Cognitively impaired (n=23)		*P* value	Overall^a^ (N=60), mean (SD)
			Mean (SD)	Median	Mean (SD)	Median		
**Time spent on the virtual reality module**														
	Time (minutes)		19.1 (3.6)	17	20.4 (3.4)	18	.17	19.6 (3.6)
**Feedback score from the questionnaire**														
	**Mean feedback score by question^b^**													
		Question 1	3.76 (1.01)	3	3.87 (0.97)	3	.67	3.80 (0.99)
		Question 2	4.11 (0.77)	4	4.13 (0.87)	4	.92	4.12 (0.80)
		Question 3	4.08 (0.76)	4	4.17 (0.94)	4	.68	4.12 (0.83)
		Question 4	3.92 (1.04)	3	4.26 (0.81)	4	.18	4.05 (0.96)
		Question 5	4.30 (0.81)	4	4.26 (0.69)	4	.86	4.28 (0.76)
		Question 6	4.51 (0.56)	4	4.43 (0.95)	4	.72	4.48 (0.72)
	Total feedback score^c^		24.7 (3.6)	25	25.1 (3.9)	26	.62	24.8 (3.7)

^a^Overall mean values from both study groups.

^b^Description of the individual questions can be found in Textbox 1.

^c^Sum of the numerical scores (ranging from 1 to 5) given for each of the six questions.

### Secondary Aim: Performance and Validity

#### Discriminating Performance Between Cognitively Intact and Cognitively Impaired Participants

In general, the mean scores for each task was higher in the cognitively intact compared to the cognitively impaired participants, except for task 1 (Table 4). These differences were statistically significant for tasks 2, 3 and 7, and the total performance score (sum of task 1 to task 7 scores) exhibited statistically significant differences. Results of the individual performance scores in the RE@CH assessment module are summarized in Table 4.

**Table 4:** Performance scores on the RE@CH assessment module by cognitive status for individual tasks and total scores.

Task	Cognitively intact (n=37), mean (SD)	Cognitively impaired (n=23), mean (SD)	*P* value
1—Opening door	96.6 (14.6)	96.7 (11.4)	.97
2—Making phone call	67.6 (33.3)	41.3 (23.4)	.002
3—Reading newspaper and identifying specific components	87.8 (20.9)	65.2 (26.9)	<.001
4—Sorting items	81.1 (19.0)	71.7 (29.5)	.18
5—Choosing the right clothes	66.9 (25.0)	57.6 (23.2)	.16
6—Withdrawing money at ATM^a^	53.4 (10.5)	50.0 (0.0)	.06
7—Shopping at supermarket	98.6 (5.7)	93.5 (11.2)	.049
Total performance score^b^	552.0 (57.2)	476.1 (61.9)	<.001

^a^ATM: automated teller machine.

^b^Sum of individual scores (ranging from 0 to 100) in each of the seven tasks.

#### Correlation of the RE@CH Assessment Module and Routine Cognitive Screening Assessment

The total performance score showed moderate positive correlation with scores from the other routine cognitive screening assessment tools, namely, AMT (0.312, *P*=.02), MMSE (0.373, *P*=.003), and MoCA (0.427, *P*<.001). In addition, the correlation of the total performance score with age was negative and poor (–0.291, *P*=.02). Table 5 presents the correlation matrix between the different variables.

**Table 5:** Correlation matrix demonstrating the degree of association among ADL score, iADL score, AMT score, MMSE score, MoCA score, age, education, total feedback, and total performance scores.

		ADL^a^	iADL^b^	AMT^c^	MMSE^d^	MoCA^e^	Age	Education	Feedback score	Performance score
**ADL**										
	Pearson correlation coefficient	—^f^	–0.01	–0.04	0.30	0.12	–0.40	0.07	0.20	–0.03
	*P* value	—	.94	.79	.02	.35	.002	.61	.13	.85
**iADL**										
	Pearson correlation coefficient	–0.01	—	0.22	0.30	0.49	–0.23	0.003	–0.03	0.20
	*P* value	.94	—	.09	.02	<.001	.07	.98	.79	.13
**AMT**										
	Pearson correlation coefficient	–0.04	0.22	—	0.73	0.73	–0.22	0.37	0.05	0.31
	*P* value	.79	.09	—	<.001	<.001	.10	.00	.68	.02
**MMSE**										
	Pearson correlation coefficient	0.30	0.30	0.73	—	0.76	–0.31	0.57	0.07	0.37
	*P* value	.02	.02	<.001	—	<.001	.02	<.001	.62	.00
**MoCA**										
	Pearson correlation coefficient	0.12	0.49	0.73	0.76	—	–0.23	0.40	–0.07	0.43
	*P* value	.35	<.001	<.001	<.001	—	.08	.002	.59	<.001
**Age**										
	Pearson correlation coefficient	–0.40	–0.23	–0.22	–0.31	–0.23	—	–0.13	–0.07	–0.29
	*P* value	.002	.07	.10	.02	.08	—	.33	.59	.02
**Education**										
	Pearson correlation coefficient	0.07	0.003	0.37	0.57	0.40	–0.13	—	–0.09	0.24
	*P* value	.61	.98	.003	<.001	.002	.33	—	.48	.06
**Feedback score**										
	Pearson correlation coefficient	0.20	–0.03	0.05	0.07	–0.07	–0.07	–0.09	—	–0.02
	*P* value	.13	.79	.68	.62	.59	.59	.48	—	.85
**Performance score**										
	Pearson correlation coefficient	–0.03	0.20	0.31	0.37	0.43	–0.29	0.24	–0.02	—
	*P* value	.85	.13	.02^g^	.003^g^	<.001^g^	.02^g^	.06	.85	—

^a^ADL: activities of daily living.

^b^iADL: instrumental activities of daily living.

^c^AMT: Abbreviated Mental Test.

^d^MMSE: Mini-Mental State Examination.

^e^MoCA: Montreal Cognitive Assessment.

^f^Not applicable.

^g^Significance at *P*<.05.

#### Receiver Operating Characteristic Curve Analysis

**Figure 4:**
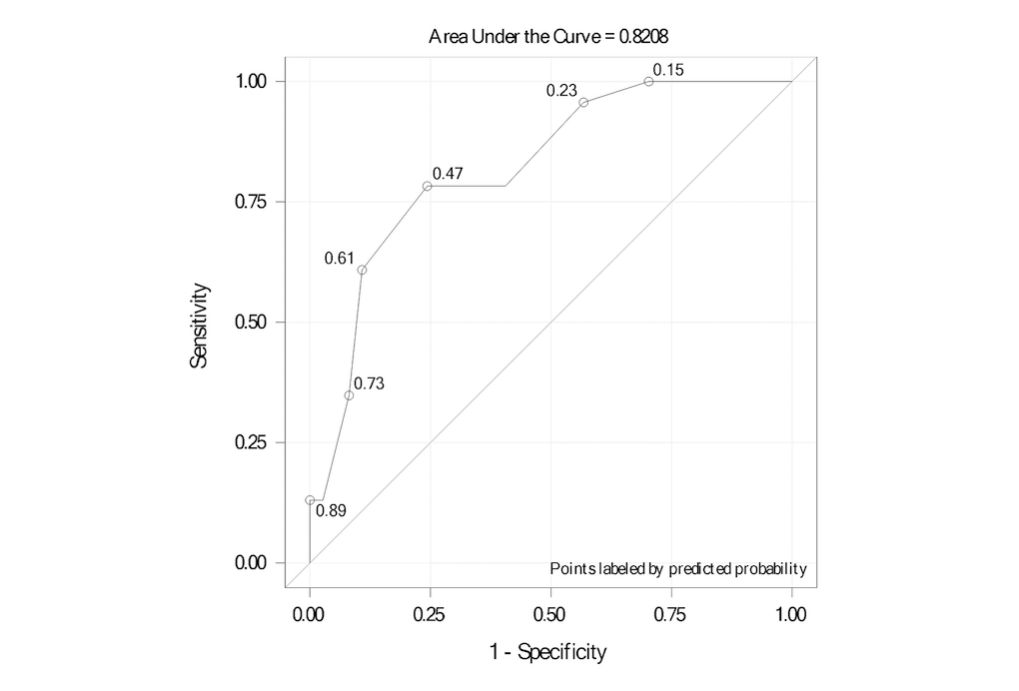
Receiver operating characteristic curve for the RE@CH assessment module’s ability to discriminate between the two groups through the total performance scores.

**Figure 5:**
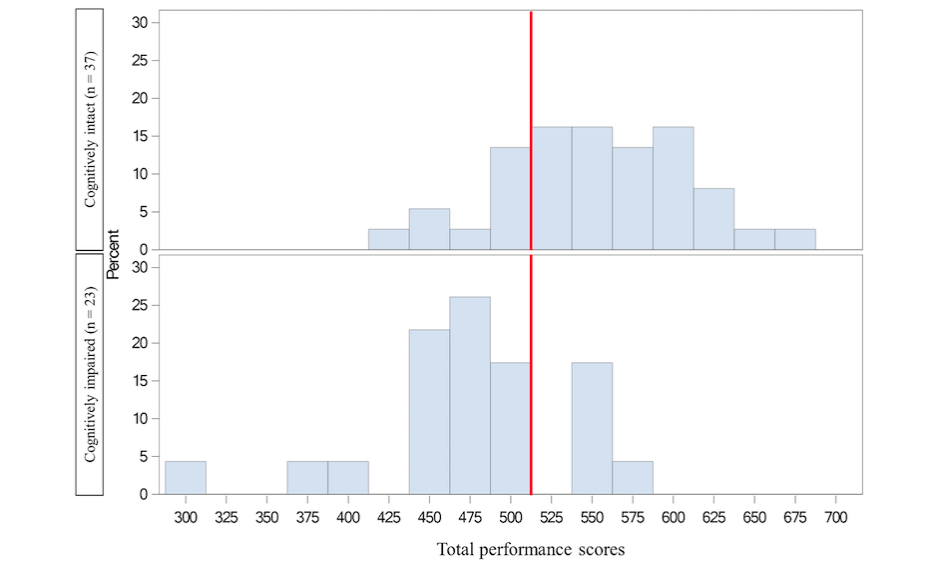
Distribution of the total performance scores of the RE@CH assessment module by group. The red line indicates the optimal cut-off score of 500.

ROC curve analysis (Figure 4) was conducted over the continuous total performance score to assess its capability to discriminate between cognitively intact (MoCA≥26) and cognitively impaired individuals (MoCA<26). The area under the curve (AUC) was found to be 0.821 (95% CI 0.714-0.928). An optimal statistical cutoff is achieved at a cut-off score of 500 (78.2% sensitivity, 75.7% specificity, 66.7% positive predictive value, and 84.8% negative predictive value; Figure 5).

## Discussion

### Primary Aim: Feasibility and Acceptability

#### Assessing Feasibility of the Virtual Reality Module in Primary Care

The primary aim of this study was to determine the feasibility and acceptability of VR to screen for cognitive impairment among older persons in primary care. We found the RE@CH assessment module feasible for this setting. All participants recruited successfully completed the study, regardless of their performance score. There were no withdrawals or any immediate adverse effects reported. The time to completion was comparable to the total time to completion of all three of the routine cognitive screening tools (AMT, MMSE, and MoCA) [5] used at SingHealth Polyclinics-Outram for memory-related complaints. Most of the participants agreed that the time spent on the VR was acceptable, as supported by a mean feedback score of 3.8 of 5 for Question 1 (Table 3).

#### Assessing Acceptance of Virtual Reality Among Participants

The RE@CH assessment module was well accepted by both participant groups, as evidenced by the positive feedback scores across all six questions. The response rate of 43.8% in this study suggests that almost one in two older persons are receptive to the use of novel technology for assessing their cognitive function. The scores for immersion in the VR experience (Table 3) were overall above average. This was supported by earlier studies that showed that older adults generally have a positive attitude toward the VR environment [21] and new technology [22]. A few felt that the VR software was not easy to use, but this could be because of unfamiliarity with VR technology.

### Secondary Aim: Performance and Validity

#### Comparing Performance Between Cognitively Intact and Impaired Participants

The secondary aim of this study was to determine the performance and validity of the RE@CH assessment module in screening for cognitive impairment. We found that although the total performance scores were significantly higher in the cognitively intact group, it was attributed to a few individual tasks. Tasks 2, 3, and 7 were better at discriminating between the two groups. The remaining tasks could be affected by the technical limitations inherent in the VR module and the structure of the scoring algorithm. Tasks 4 and 5 were challenging to both groups, because the motion sensor was not precise enough to pick up all hand gestures. Tasks 1 and 6 were relatively easy, and all participants performed well. This might have led to underperformance or overperformance across the groups, but it is not likely to have differentiated between the two groups well.

#### Assessing Validity of the Virtual Reality Module Compared to Other Screening Assessments

The RE@CH assessment module generated valid total performance scores that had a moderate positive correlation with all the other three validated cognitive screening assessments. A high positive correlation was not expected, since the latter focused specifically on a few cognitive domains and the VR module could have identified cognitive deficits that other screening assessments were unable to detect. The ROC analysis indicated relatively strong prognostic classification capability [23] with an AUC of 0.821 when benchmarked against MoCA scores.

### Clinical Implications

Our findings provide preliminary evidence that VR modalities, such as the RE@CH assessment module, can be used for cognitive screening among older person in primary care. We built upon the positive results from previous studies [11-13,24] and introduced another integral component of a VR setup—the Leap motion sensor—that detects hand gestures [25]. Instead of a single game, we introduced multiple short VR-based activities to assess the different cognitive domains.

Our findings show that participants could still perform most of the tasks within a time frame of about 20 minutes (mean 19.6 minutes). In addition, we showed that VR was not only feasible, but also relatively practical to execute within the premise of a primary care clinic. The screening can be executed prior to consultations with the primary care physician to optimize their clinic visits by at-risk patients. Implementation studies are needed in future research to assess its roll-out in clinical practice.

### Limitations

This study had several limitations. First, there was a significant difference in the level of education between the two groups. More participants with MoCA scores≥26 had higher educational levels than those with lower MoCA scores, which could impact the outcomes of their performance. The disparity could be related to the recruitment process. Participants who were better educated and more likely to have been exposed to health technology and innovations were likely and willing to be enrolled into the study, even in the absence of cognitive symptoms. The sample size was small in this feasibility study. An adequately powered randomized controlled trial, stratified by education level, is planned to further assess the use of VR in cognitive assessment.

Second, the participants might not be representative of the entire target population at risk of cognitive impairment (older persons aged≥65 years) [26], since recruitment was carried out only at one location with multiple exclusion criteria. Furthermore, the study population was classified solely on the basis of the MoCA scores, which is a screening tool and not considered diagnostic of cognitive impairment. Participants with subjective cognitive impairment but normal MoCA scores could have been assigned erroneously to the cognitively intact group.

Lastly, the RE@CH assessment module was a prototype that was originally used for rehabilitation and then adapted for cognitive screening. It included tasks that might be challenging to complete because of the prototype content design, and not due to the participant’s cognitive impairment. A specially designed VR program that will be developed by us to assess cognitive function has been recently awarded funding by a local public information technology agency, which will address the limitations of the current prototype. The next prototype will eliminate tasks that do not differentiate between the two groups to both reduce redundancy and optimize the time spent to complete the cognitive assessment. The next study will include time motion measurements to evaluate accuracy, efficiency, and cost-effectiveness of our tool in comparison with the conventional screening tools such as MoCA.

### Conclusions

The study successfully demonstrated the feasibility of a VR-based screening tool in primary care. The target population expressed a positive perception of and attitude toward VR and were open to the use of this type of technology for their cognitive assessment. The results of this feasibility study are invaluable in the design of a novel VR program and study protocol by validating its use as a comprehensive, multidomain cognitive function screening tool in the next phase of development.

## Abbreviations

ADL: activities of daily living
AMT: Abbreviated Mental Test
AUC: area under curve
iADL: instrumental activities of daily living
GRACE: GeRiAtric serviCE
MCI: mild cognitive impairment
MMSE: Mini-Mental State Examination
MoCA: Montreal Cognitive Assessment
PI: primary investigator
ROC: receiver operating characteristic
VR: virtual reality
